# Importance of assessing cytogenetic and molecular risk factors in acute myeloid leukemia therapy

**Published:** 2012

**Authors:** EC Coles, A Colita, R Momanu, N Berbec, AM Ivanescu, M Oprea, D Jardan, C Jardan, A Arghir, D Coriu, AR Lupu

**Affiliations:** * Hematology Department, Coltea Clinical Hospital, Bucharest; ** Roche Romania, Bucharest; *** “Carol Davila” University of Medicine and Pharmacy, Bucharest; **** Fundeni Clinical Institute Bucharest; ***** Victor Babes Institute Bucharest

**Keywords:** Acute myeloid leukemia, cytogenetic abnormalities, molecular anomalies, complete remission, prognosis, **AML-** acute myeloid leukemia, **APL**- acute promyelocytic leukemia, **CR**- complete remission, **ATRA**- all trans retinoic acid, **ASCT**- allogeneic stem cell transplant, **OS**- overall survival, **DFS**- disease free survival, **EFS**- event free survival, **LdAC**- low dose Ara-C, **Ara-C**- Cytarabina, **FAB**- french- american- british, **MRD**- minimal residual disease

## Abstract

Acute myeloid leukemia (AML) is a heterogeneous disease in clinical presentation, outcome and therapeutic response. Cytogenetic and molecular characteristics are important prognostic indicators allowing the identification of distinct subtypes of AML, prognostic stratification and risk-adapted treatment.

We present our experience during 5 years, in which we treated 245 patients with AML, of which we could genetically characterize 48 cases (26 females, 22 males) with a median age of 52 years.

Cytogenetic analysis was performed by GTG banding on cultures of marrow cells treated with colcemid. Molecular analysis used RT-PCR performed on ABI 9700 platform in order to identify the following fusion genes: E2A-PBX1, TEL-AML1, AML1-ETO, PML-RARα, MLL-AF4, CBFC-MYH11, BCR-ABL, SIL-TAL, and MLL-AF9as well as mutations in Flt3, NPM1, WT1 genes.

Fourteen patients were older than 60 years. In 12 we performed cytogenetic analysis showing 5 cases with complex karyotype, 2 normal karyotypes, 1 case of del(21), del (9), 11q- and t(3;15) respectively as well as 2 unevaluable karyotypes. These anomalies were associated with a high incidence of secondary AMLs (10/14) and with a low remission (CR) rate (5/14).

Out of the 35 patients younger than 60 years, 25 were evaluated by cytogenetics showing a high incidence of favorable cytogenetic changes: 6 anomalies of chromosome 16 (5 inv (16) and 1 t (16; 16)), 3 t (15; 17), 3 cases of t (8; 21) of which 2 with additional abnormalities, 7 normal karyotypes and 1 case of 7q-, -y,-3 and respectively -8 associated with +18. In 25 cases molecular analysis was performed showing alterations in 21 patients: 6 cases with AML/ETO, 3 PML/RAR, 7 Flt3 mutations (2 associated with NPM1 mutation) as well as 1 case of isolated mutation of NPM1 and respectively WT1. CR rate was of 28/35. All cases with t (15; 17) and PML/RAR as well all cases with t (8; 21) and/or AML/ETO achieved CR. Out of the 7 cases with Flt3 mutations only 4 achieved CR including the 2 cases with associated NPM1 mutations.

In our experience, genetic characteristics correlate with other prognostic markers such as age and secondary leukemia; “favorable” genetic anomalies were associated with a high CR rate; association of t (8; 21) with additional abnormalities did not influence CR rate.

## Introduction

Acute myeloid leukemia (AML) is a heterogeneous disorder in terms of clinical presentation, evolution and response to treatment.

Marrow and peripheral morphological assessment remains of reference, but cytogenetic and molecular characteristics represent extremely important prognostic factors, which allow for the identification of distinct subtypes of AML, prognostic stratification and risk groups adapted therapy [**1**].

Karyotype analysis plays a crucial role in characterizing AML, in determining the aggressiveness of the disease, the response to treatment and the prognosis [**13**]. Favorable cytogenetic abnormalities (inv16, t(16;16), t(15;17), t(8;21)), intermediate (normal karyotype, +8, del (9q), del (7q)) or of reserved prognostic (del (5q)), -5, -7, complex karyotype) are among the most valuable independent prognostic factors, and thus they may lead to an adapted therapeutic approach. APL with the presence of t(15;17), treated with ATRA and anthracycline, or AML with presence of t(8; 21)(q22, q22), inv(16) (p13; q22) / t(16;16) treated with intensive chemotherapy, are characterized by high rates of CR and the relapse incidence is much lower, so that ASCT in first remission shows no benefit in terms of survival (OS) [**10-12**]. Instead, the presence of 3q, 5q-, or complex karyotype, gives a very reserved prognosis to conventional chemotherapy and thus patients are considered candidates for allogeneic transplantation or clinical trials [**7-11**].

Chromosomal aberrations are not detected in 40-50% of patients [**1,2,29**]. The presence of molecular abnormalities in patients with normal karyotype allows for risk stratification, the evolution prediction and therapy modulation [**3-6**].

Detection of molecular markers such as NPM1, FLT3, CEBPA has become a routine [**1**]. Instead, mutations in WT1, IDH1/IDH2, TET2, RUNX1, MLL genes or aberrantly expressed genes (BAALC, ERG, EVI1) will be useful for better assessment of molecular risk [**1**].

## Patients and methods

The trial was conducted over a period of 5 years (October 2007-October 2012), on a lot of 243 cases with AML, treated in Coltea hematology clinic. Cytogenetic and / or molecular analysis was performed in 48 cases (26 women, 22 men), mean age was 52 years. Cytogenetic analysis was performed using bone marrow cell cultures treated with colcemid to be blocked in metaphase, and GTG banding in the Genetic Laboratory of Fundeni and Babes Institutes.

For the molecular analysis we used method RT-PCR on ABI 9700 platform to identify fusion genes: E2A-PBX1, TEL-AML1, AML1-ETO, PML-RARα, MLL-AF4, CBFC-MYH11, BCR-ABL, SIL-TAL , MLL-AF9 and mutations of genes FLT3, NPM1, WT1. – Molecular Biology Fundeni Institute.

CR induction therapy protocols consisted of three regimens, namely “7+3” (Cytozar 100mg/m2 -7 days and Doxorubicin 45mg/m2- 3 days), AIDA (ATRA 45mg/m2 and Idarubicin 12mg/m2 in day 2-4-6-8) and ldAC (Ara-c 20mg/day).

## Lot features

Patients evaluated from a cytogenetic and molecular point of view were between 18-80 years with a median age of 52 years, patients under 60 years being predominant.

Most cases were AML "de novo", about 2/3 of cases (especially young patients), the rest being AML post myelodysplastic syndrome or secondary to chemotherapy / radiation therapy (predominantly in the elderly group).

The parameters used to evaluate the lot of patients were multiple: signs and symptoms at onset (fatigue, bleeding syndrome), infections, associated comorbidities / neoplasia, peripheral and bone marrow blasts percentage, FAB subtype, remission rate (after one / several induction course), CR duration, relapse (early / late).

**Table 1.** The parameters used to evaluate the lot of patients

**Lot features**	
Age - years	18-80 (median 52)
> 60	14
< 60	34
AML Type	De novo – 35 cases
	Secondary (SMD/CT) - 13
Hb (g/dl)	4,1 – 12,4 (median 8)
L (109/L)	0,4 – 223 (median 13,1)
Tr (109/L)	0 – 290 (median 31,5)

## Results

Response to treatment

**Table 2.** Complete remission rate

	
CR Rate	29 cases (60,4%)
CR Duration	1-53 months (median 14 months)
CR Rate < 60 years	79%
> 60 years	28%
Rate ~ AML Type- de novo	66%
- secondary	46%

All patients younger than 60 years (34 cases) underwent aggressive chemotherapy ("7 +3" AIDA), and after the first induction complete remission (CR) was obtained in 13 patients (38.2%), from whom 3 patients were with APL. In elderly group (14 patients) "7+3" was performed only in case of 3 patients (21.4%) (good performance status) and after the first induction CR was obtained in 2 patients (chromosome 9 abnormalities), of which 1 case with AML postchemotherapy for gastric adenocarcinoma. In the remaining patients aged over 60 years, Ara-C was performed in small doses, after the first induction CR was obtained only in 1 patient.

### Treatment results by cytogenetic characteristics

Cytogenetic examination revealed in elderly patients a predominance of complex karyotype (approximately 50%), while in case of the younger group, normal karyotypes or chromosomal aberrations with favorable prognosis were found predominantly.

In the lot of patients younger than 60 years who had performed cytogenetic analysis in 25 cases, the presence of favorable translocations (t (15;17), t (8;21), inv (16)) resulted in CR in all cases, which was maintained over a significant period of time(over 24 months) for APL with presence of t (15;17). Loss of chromosome 3 or deletion of 7q- was associated with treatment failure. In particular, cases of trisomy 8 associated to monosomy 18, and sex chromosome loss were chemosensitive, with a duration of CR of almost 12 months.

**Table 3.** CR rate according to chromosomal anomalies in the lot of patients < 60 years old

**Karyotype**	**Number of cases**	**CR**	**CR Duration Median (months)**
Normal	7	5	3
Abnormalities Cz16 (5 inv16; 1 t(16;16))	6	5	4
t (15;17)	5	5	27
t (8;21) (+ AA)	3(2)	3	5
7q-	1	0	-
-Y	1	1	12
- 3	1	0	-
+8 associated -18	1	1	9

In case of the lot over 60 years, who underwent cytogenetic examination in all cases, normal or complex karyotype was not associated with therapeutic response. CR was obtained in all other cases, but it did not last (relapse was early).

**Table 4.** CR rate according to chromosomal aberrations in the lot > 60 years

**Karyotype**	**Number of cases**	**CR**	**CR Duration (months)**
Normal	2	0	-
Complex	5	0	-
-21	1	0	-
-9	1	1	10
11q-	1	1	2
t (3;14)	1	0	-
Inv (9)	1	1	1
Non-assessable	2	1	2

Chromosomal abnormalities were detected in 28 (71.8%) of patients who underwent karyotype examination, the remaining 11 (28.2%) having normal karyotypes or non-assessable. Aberrations detected were, in order of frequency, chromosome 16 abnormalities (6 cases-15, 3%), t (15;17) (5 cases-12, 8%), complex karyotype (5 cases-12, 8%), t (8;21) (AA) (5 cases-12, 8%), abnormalities of chromosome 9 (2 cases-5, 1%), and 1 case (2.5%) of 7q-,-Y, -3, +8 associated -18, -21, 11q-, t(3;14).

### Results of treatment according to molecular characteristics

Molecular analysis in the lot with patients younger than 60 years old showed a significantly higher proportion of anomalies with favorable prognosis (6 cases AML-ETO, PML-RARA 5 cases, 1 case NPM1) compared to the presence of mutations with poor prognosis (5 cases FLT3 -ITD, 2 cases FLT3-ITD + NPM1, 1 case with mutations in WT1 gene).

In the group older than 60 years, 2 cases showed no detectable abnormalities, one case presented FLT3 gene mutations and one MLL gene mutations.

In the analyzed group of 34 patients younger than 60 years, we performed molecular analysis in 20 cases.

In all the cases that revealed favorable molecular abnormalities, we obtained complete remission, which was maintained for a period of approximately 18 months.

Although in most cases in which abnormalities with poor prognosis were detected therapeutic response was achieved, early relapse rate was high (CR median was 1 month).

**Table 5.** CR rate according to molecular anomalies in the lot < 60 years

**Molecular biology**	**Number of cases**	**CR**	**CR Duration Median (months)**
AML/ETO	6	6	18
PML/RARα	5	5	20
FLT3 – ITD	5	2	1
FLT3 – ITD + mut NPM1	2	2	1
NPM1	1	1	12
WT1	1	1	1

In the analyzed group, of 14 patients older than 60 years, we performed molecular analysis in four cases: in 3 cases we obtained therapeutic response (1 case with MLL gene aberrations and 2 cases which showed no detectable abnormalities), 1 case with FLT3 gene mutations that resulted in treatment failure.

**Table 6.** CR rate according to molecular anomalies in the lot > 60 years

**Molecular biology**	**Number of case**	**CR**	**CR Duration Median (months)**
FLT3	1	0	0
MLL	1	1	1
No mutations	2	2	2

After the results of the molecular biology, we divided patients into the favorable lot (12 cases-50%), the negative lot (10 cases-41, 6%) and the lot without detectable mutations (2 cases-8, 4%).

## Discussions

The results of this trial, otherwise not on a very significant lot in number, are comparable with other trials and come to strengthen existing statistics.

CR rate after the first induction, independent of regimen or age, was 33.3%.

CR rate was ~ 80% in the younger group, and of over 25% in patients over 60 years.

Chromosomal aberrations were noted in 71.8% of cases.

Favorable cytogenetics abnormalities were revealed only in younger patients and were accompanied by a CR rate of 81.2%.

In elderly patients, neither normal karyotypes nor favorable molecular abnormalities were revealed, as the complex karyotypes or negative molecular aberrations were the predominant ones.

The molecularly favorable lot (exclusive young patients) had a CR rate of 100%, with a median of 12 months. In particular, the molecularly negative group had a high rate of CR (60%), but resulted in early relapse in all cases.

AML de novo was seen especially in the young group.

AML secondary to chemotherapy / myelodysplasia was evident mainly in the elderly group, being accompanied in all cases by therapeutic failure.

Response to chemotherapy, although we obtained it both in case of cytogenetic aberrations and of molecular abnormalities with poor prognosis, was not supported.

It was not possible to calculate accurate rates of DFS or OS because of the absence of a rigorous follow-up.

To further clarify these results, which have statistical value, a larger trial on a bigger lot of patients and a more rigorous follow-up would be required.

Karyotype examination remains one of the most significant prognostic indicators in AML [**1**]. Multicenter studies revealed that APL with presence of t (15;17)/PML-RARA, or those with t(8;21) / RUNX1-RUNX1T1/AML1(21q22)-ETO(8q22) with or without additional abnormalities (del (9)(q22)) or with inv (16)/t(16;16)/CBFB-MYH11 is accompanied by positive developments, a good response to chemotherapy, a high rate of remission and prolonged DFS [**1,2**]. On the other hand, the presence of abnormalities of chromosome 5 (del (5q), -5), of chromosome 7 (-7), complex karyotype are associated with an adverse prognosis [**1,2**]. Sex chromosome loss, until recently considered a non-fenotipical event occurred in elderly men, is now revealed as a marker for the leukemic clone and associated with an unfavorable evolution [**14,16**].

NPM1 gene mutations are the most common abnormalities acquired in AML [**17,18**]. And are closely correlated with a high number of white blood cells, with normal karyotype, and FLT3-ITD mutations [**24,25**]. Patients with intermediate cytogenetic risk, with the absence of FLT3-ITD, but with NPM1 presence, show higher rates of OS and EFS and a reduced incidence of relapse than patients in whose case NPM1 has not been detected [**19,25,26**]. It was not obvious from the beginning whether NPM1 is a primary genetic alteration and whether additional chromosomal aberrations and multilineal dysplasia had any impact on biological and prognostic features of AML [**22**]. Studies have shown, however, that the presence of NPM1 imprint AML clinical and biological features, as a reduced expression or absence of CD34, and seems to be an initiating genetic event in approximately 1/3 of cases of AML, and thus extremely useful in determining minimal residual disease [**21-24**]. Besides the NPM1 mutation, only age and FLT3 mutation status are significantly prognostic for EFS [**24**].

Other studies that included age, FLT3 status and NPM1 level at different time points, have concluded that the NPM1 level was the most relevant prognostic factor during the first-line treatment, or second-line chemotherapy or in ASCT case [**20,24**]. Also, the independent expression of NPM1 has favorable prognosis level in terms of response toward treatment, OS, EFS and DFS [**19,24,25**].

FLT3 mutations are among the most common molecular abnormalities detected in patients with AML [**29,31,32**]. Patients with presence of FLT3-ITD but with absence of FLT3-TKD have a significantly more reserved prognosis [**32**]. Fms-like tyrosine kinase is a receptor of tyrosine kinase involved in the regulation of proliferation, differentiation and apoptosis of hematopoietic cells [**1,15**]. Its mutations are present in 1/3 of patients with AML [**27,30,32**]. Activating FLT3 by internal tandem duplication (ITD) is one of the most common molecular alterations in AML and was observed in SMD as well before and during progression towards AML [**29,31**]. The presence of FLT3-ITD confers an increased risk of relapse and death; when detected in elderly patients it can predict the worse outcome [**33,34**]. In recent years, FLT3 is emerging as a promising molecular target in AML therapy [**28-32**].

WT1 (Wilms tumor antigen) is involved in leukemogenesis and is overexpressed in all types of leukemia [**35**]. Recent studies reveal its role in the formation of the haematopoietic system [**36**]. Independently expressed WT1 gene mutations are correlated with a poor prognosis [**35**]. But mutation status might change in some patients during AML evolution, as it can disappear at CR achievement, which gives it great utility in detecting BMR [**37**].

MLL gene mutations are especially associated with AML secondary to chemotherapy / radiotherapy and ALL in children, thus predicting a poor prognosis.

## Conclusions

Current therapeutic approach in AML involves a pre and post treatment complex evaluation. Before any therapy, age, performance status, and especially cytogenetic and molecular evaluation, can greatly determine the therapeutic conduct.

The usefulness of cytogenetic examination may be limited when the karyotype is non-assessable or normal (approximately 40% of patients). In these cases, molecular biology can modulate treatment by the detection of abnormalities for which molecular therapies are targeted.

Treatment response was revealed as an independent prognostic factor. Using RQ-PCR in MRD detection is based on detection of molecular targets (fusion genes PML-RARA, RUNXIT1-RUNX1, CBFB-MYH11) or of mutations (NPM1 mutation) or of overexpressed genes (WT1).

A constant challenge is the therapeutic attitude in AML in the elderly patients, who appear to have distinct clinical and biological features that translate into reduced chemosensitivity (CR rate <45% compared with 75% in younger patients), treatment-related mortality (~ 25%) median survival (~ 10 months) and low rate of OS (10 months) [16]. Karyotype evaluation can optimize treatment, especially in these patients, for whom the cytogenetics prognosis is generally poor (abnormalities of CRZ 5, 7, 8, or complex karyotype) [17]. The presence of favorable chromosomal aberrations could allow a more aggressive therapeutic approach in case of an elderly patient with good performance status.

Response to therapy, the remission duration, evolution (DFS, OS) can be improved by combining pre-and post-treatment parameters in a prognostic algorithm. While chromosomal and molecular abnormalities may fail to predict the individual evolution of each patient, measuring minimal residual disease (MRD) is now recognized as a parameter by which we can assess the quality of response after chemotherapy, and based on which we can adopt post-remission strategies and predict an accuracy prognosis.

## Acknowledgement

This paper is partially supported by the Sectoral Operational Programme Human Resources Development, financed from the European Social Fund and by the Romanian Government under the contract number POSDRU/89/1.5/S/64153
